# Measuring public attitudes towards people living with chronic diseases in Arabic-speaking populations: adaptation and development of the Social Stigma Scale of Chronic Diseases (SSS-CD)

**DOI:** 10.1186/s12889-023-16315-1

**Published:** 2023-07-18

**Authors:** Feten Fekih-Romdhane, Sahar Obeid, Georgio Chidiac, Mariam Dabbous, Diana Malaeb, Rabih Hallit, Souheil Hallit

**Affiliations:** 1 The Tunisian Center of Early Intervention in Psychosis, Department of Psychiatry “Ibn Omrane”, Razi hospital, Manouba, 2010 Tunisia; 2 Faculty of Medicine of Tunis, Tunis El Manar University, Tunis, Tunisia; 3 Social and Education Sciences Department, School of Arts and Sciences, Lebanese American University, Jbeil, Lebanon; 4 School of Medicine and Medical Sciences, Holy Spirit University of Kaslik, P.O. Box 446, Jounieh, Lebanon; 5 Department of Dermatology, University Claude Bernard Lyon 1, Lyon, France; 6 School of Pharmacy, Lebanese International University, Beirut, Lebanon; 7 College of Pharmacy, Gulf Medical University, Ajman, United Arab Emirates; 8 Department of Infectious Disease, Bellevue Medical Center, Mansourieh, Lebanon; 9 Department of Infectious Disease, Notre Dame des Secours University Hospital Center, Street 93, Byblos, Postal Code 3, Lebanon; 10 Research Department, Psychiatric Hospital of the Cross, Jal Eddib, Lebanon; 11 Applied Science Research Center, Applied Science Private University, Amman, Jordan

**Keywords:** Chronic diseases, Social stigma, Attitudes, Psychometric properties, Arabic

## Abstract

**Background:**

A large proportion of the population in Arab countries suffer from chronic diseases. According to the statistics by the Global Health Estimates, chronic illnesses contribute by 71% to total deaths in the Arab region. While chronic illnesses have been demonstrated to carry high levels of social stigma, it is only recently that little research attention has been given to this topic in the Arab world. It is well-established that the social stigma construct is culturally-dependent. Therefore, the lack of an Arabic measure highlighted the urgent need for developing a culturally adapted and valid instrument to assess social stigma toward people living with chronic diseases. In this study, we aimed to examine the psychometric properties of the Arabic translation, adaptation and development of “the Social Stigma Scale of Chronic Diseases” (SSS-CD).

**Method:**

Fifteen items derived from the literature and assessing social stigma towards chronic diseases have been administered to 570 Arabic-speaking adults from the Lebanese general population (aged 24.59 ± 6.75years; 68.6% women). Items were translated into Arabic using a forward-backward translation method. Exploratory factor analysis (EFA) using a principal-axis EFA on the first split-half subsample, followed by a confirmatory factor analysis (CFA) tested on the model extracted from the EFA on the second split-half subsample, were conducted to examine the construct validity of the SSS-CD. Fit indices were deemed adequate if the normed model chi-square (χ²/df) ≤ 3, the Steiger-Lind root mean square error of approximation (RMSEA) ≤ 0.08, the Tucker-Lewis Index (TLI) and the comparative fit index (CFI) ≥ 0.90.

**Results:**

Findings revealed that the 10-item SSS-CD has a unidimensional factor structure, with the following fit indices: χ^2^/df = 92.95/34 = 2.73, RMSEA = 0.077 (90% CI 0.059, 0.096), SRMR = 0.062, CFI = 0.939, TLI = 0.919. A good internal consistency was demonstrated by a McDonald’s omega value of 0.73 for the total score. Findings also supported invariance across gender, with men exhibiting higher levels of social stigma attached to chronic diseases than women. All three dimensions of stigmatization (social, psychological and evolutionary stigmatization) were positively correlated with SSS-CD scores (Social self-interest [r = .40; p < .001], Evolutionary self-interest [r = .37; p < .001], Psychological self-interest [r = .42; p < .001]), demonstrating relatively strong convergent validity.

**Conclusion:**

Our findings suggest that the SSS-CD has robust psychometric qualities. We thus preliminarily suggest that the scale is valid, reliable and suitable for use among Arabic-speaking people from the general population to measure public attitudes towards people living with chronic diseases. Providing this psychometrically sound measure will hopefully enable to foster research in this area in order to draw a clear overview of the prevalence and characteristics of social stigma attached to chronic diseases in Arabic-speaking communities. However, given that this was the first study to examine the psychometric properties of the SSS-CD, the present findings and conclusions should be considered tentative pending future cross-national validation studies.

## Background

Like other countries worldwide, a large proportion of the population in Arab countries suffer from chronic diseases such as cardiovascular, hypertension, and diabetes [1]; mainly because of the widespread smoking, unhealthy diet, low physical activity, and obesity [2]. Chronic illnesses represent one of the causes of high morbi-mortality among the population in the Arab region [1], contributing by 71% to total deaths according to the statistics by the Global Health Estimates [3]. For instance, a study conducted among Emirati University students [4] found a prevalence of chronic diseases of 27.5% and 21.8% in males and females, respectively, with 4.7% reporting two or more chronic diseases. In Saudi Arabia, non-communicable diseases are responsible for 73% of all deaths [5]. Another recent study [6] reported that in the Middle East and North Africa region, Parkinson’s disease had an age-standardized point prevalence of 82.6 per 100,000 people and an age-standardized mortality rate of 5.3 in 2019, both of which had increased from 1990 to 2019 by 15.4% and 2.3%, respectively.

Chronic illnesses have been demonstrated to carry high levels of shame [7–9], and stigma [10]. Stigma is an accepted negative stereotype that a community has about particular individuals. It is a universal phenomenon, defined as social discrediting or devaluation due to an attribute or a mark [11]. Stigma also refers to a discrediting feature that makes someone feel degraded and flawed in the eyes of others [12]. The idea of stigma as a social phenomenon describes the society views towards the affected person as being less valuable in the perspective of others [13]. Social stigma is thus defined as the negative perception or attitude of discrimination toward a given group (here, patients diagnosed with chronic illnesses) due to the characteristics and traits it represents [14–16]. Social stigma related to chronic illnesses leads the concerned individual to feel blamed, rejected [17], irresponsible [18, 19], ashamed, guilty, unworthy [20], responsible for their disease [21, 22], not allowed to work with children [23–26], and “trapped” in a new, different identity and life [27]. Stigma also leads people to experience workplace discrimination [28] and poor healthcare [29] because of their disease and symptomatology; thus adding to the already experienced burden of diminished physical and mental health [30]. In addition, people living with chronic illnesses are perceived as having poor prospects [31], fewer physical, emotional, economic, social resources to offer others, and as being unable to be consistently relied on [32].

Therefore, chronic diseases-related stigma has multiple detrimental effects on physical and mental health. The psychological consequences of living with a stigmatized chronic disease includes depression and overall distress [33–36], Anxiety [22], social isolation and exclusion [37], avoidance of social activities [22], and decreased quality of life [38, 39]. These negative consequences on patients’ psychological status lead in turn to lower access to healthcare [39], neglected self-care and poor clinical outcomes [36]. It is therefore paramount to address social stigma of chronic diseases and its impact on mental and physical heath for a holistic approach of patients [40]. Indeed, determining the evidence about social stigma in chronic diseases may help in advancing our knowledge beyond describing the causes and consequences of stigma experiences toward developing more informed and effective mitigating strategies. The first step toward achieving this evidence is using a measure that accurately reflects the social stigma concept. However, research on social stigma associated with the diagnosis of chronic diseases is still lacking, and hindered by the inexistence of measurement instruments assessing this specific construct [36, 41]. It is only recently that little research attention has been given to chronic diseases-related stigma in the Arab world; with most of the existing studies having examined self-perceived stigma in clinical populations (e.g., patients with psoriatic [42, 43], COVID-19 [44] Tuberculosis [45]); while fewer studies have focused on investigating the stigmatizing perceptions of the general public towards patients with chronic diseases. For instance, Tayed et al. [46] examined the perceptions of a community Saudi sample towards people with epilepsy using self-developed items previously used in an Iranian study (i.e. [47].). Another study investigated the perception of Arab community adults towards people with Alzheimer’s disease using the vignettes method, which has been discussed as a limitation of the study [48]. Soffer examined human immunodeficiency virus/acquired immune deficiency syndrome (HIV/AIDS) -related beliefs among healthy Arab women using a qualitative design methodology [49].

The existing measures were rather designed to assess anticipated/self-perceived stigma among patients with chronic diseases (e.g., the Stigma Scale for Chronic Illnesses (SSCI) [50], the Chronic Illness Anticipated Stigma Scale (CIASS) [32]). We could find only one measure that has been previously validated in Arabic, which assesses self-perceived stigma exclusively associated with COVID-19 among infected patients [51]. Regarding social stigma related to chronic illnesses in particular, the vast majority of studies used qualitative research approaches (e.g., [23, 52–55]). Other researchers opted for a self-developed single-item measure, while changing the wording for each of the concerned diseases (i.e. ‘‘What score would you rate for the prejudice that the general population has towards: epilepsy, AIDS, and diabetes’’) [56]. Studies using quantitative measures were rather illness specific, (e.g., dementia [57], COVID-19 [58], Epilepsy [59]), which do not allow for comparisons across the different chronic illnesses groups. To the best of our knowledge, none of these scales is available in the Arabic language, constraining researchers’ attempts to determine the prevalence of public attitudes towards people living with chronic diseases and its contribution to adverse health and psychological effects. A reliable, validated Arabic tool would allow researchers to measure the impact of chronic diseases-stigma interventions on other health outcomes [60]. However, the experience of stigma across the different chronic illnesses has demonstrated large similarities [41]; or even unexpected differences (e.g., people living with hypertension displayed significantly greater perceived and internalized stigma than those living with HIV [61]). This has led some researchers to recommend the design of ‘generic’ measures of stigma to avoid duplicated efforts across disciplines [41, 62]. This emphasizes the strong need for valid and reliable measures to investigate chronic diseases-related social stigma in both community and research settings. Additionally, the culturally-dependent nature of the social stigma construct [63, 64], and the lack of an Arabic measure, highlighted the urgent need for developing a culturally adapted and valid instrument to assess social stigma toward people living with chronic diseases. Adding to that, the growing prevalence and impact of chronic diseases continues to challenge health systems worldwide, and effective national chronic diseases policies and strategies have been demonstrated to reduce this burden of chronic diseases [65]. Therefore, it’s deemed necessary to assess public attitudes towards people living with chronic diseases and consequently provide educational campaigns for the general population and programs to the at-risk population to promote healthy behaviors and avoid modifiable risk factors of chronic diseases [66]. Hence, understanding the stigma towards people living with chronic diseases could then guide efforts to address the most pressing domains of stigma and in turn remove barriers to chronic diseases prevention, care and treatment [67].

In this study, we chose to translate to Arabic, adapt and validate one scale that has been designed by Green et al. [68] to assess social stigma attached to HIV. The scale is composed of 15 items worded to target people with HIV (e.g., “People with HIV should be ashamed of themselves” or “People with HIV are not to be trusted”). Items are scored on a 4-Likert point scale ranging from “strongly disagree” to “strongly agree”. In the original validation, authors found that a one-factor solution produced the best fit to the data, and was more reliable than the sub-scales based upon the three separate theoretically-based domains (i.e., cognitive aspects, victim-blaming, and treatment from society). After an extensive, in-depth literature review, this scale was deemed appropriate to measure social stigma attached to any other chronic disease, and this, for many reasons (further details about the development and adaptation of the scale can be found later in this paper). Levels of stigma held by the general public toward people with HIV has been found to be close to those exhibited toward people with epilepsy [69], and even lower in certain aspects than stigma toward people with hypertension [61]. Additionally, stigma associated with HIV and other chronic diseases share multiple characteristics. Indeed, people fear chronic diseases of any type and attribute its onset to undesirable characteristics of the affected person and blame them, which gives them the illusion of control and feelings of protection against vulnerability [70, 71]. This might lead to aversion of others toward people living with chronic diseases, and in turn to social exclusion/rejection, and reluctance to disclose the diagnosis (e.g., HIV [72], cancer [73], diabetes [74], epilepsy [75], tuberculosis [76], dementia [77]). People with HIV, as well as with other chronic diseases, may be perceived as responsible for their diseases (HIV, severe acute respiratory syndrome (SARS), and tuberculosis [78]; cancer [17]; diabetes [36], obesity [79]), ashamed on themselves (HIV [80], tuberculosis [81], epilepsy [82], diabetes [83, 84], cancer [85], obesity [86], psoriasis [87]), untrustworthy (HIV [88], chronic pain [89], scleroderma [90], non-epileptic seizures [91], diabetes), less intelligent (HIV [92], Diabetes [74], obesity [79], epilepsy [93]), weak-willed (HIV [68], chronic pain [89], cancer [94], diabetes [95], obesity [96], migraine [97]), and not allowed or unable to play or work with children (HIV [98], epilepsy [93], diabetes [99], cancer [100]).

For all these reasons, and for the purposes of this study, the word “HIV” in each item of the above-mentioned scale was changed to “chronic diseases” (e.g., “People with chronic diseases should be ashamed of themselves” or “People with chronic diseases are not to be trusted”), in order to assess social stigma held by society toward people living with different chronic illnesses; and the new, modified version of the scale was labeled as “the Social Stigma Scale of Chronic Diseases” (SSS-CD). Thereafter, the psychometric properties of the Arabic translation of the SSS-CD were examined. We hypothesized that the scale will confirm the originally proposed one-factor structure identically in both genders and will show a good reliability. Additionally, convergent validity is expected to be evidenced by testing whether the SSS-CD relates to another measure of Social Stigmatization, i.e., The Standardized Stigmatization Questionnaire (SSQ) [101], which assesses perceived social stigmatization (i.e., how respondents think most people will react to a person who becomes ill), and predisposition to enact stigmatization towards the ill person. As both the SSS-CD and the SSQ measure the same construct of social stigma, we hypothesized that SSS-CD scores will correlate positively and strongly with SSQ dimensions.

## Methods

### Procedures

All data were collected via a Google Form link, between July and August 2022. The survey needed between 10 and 15 min to be completed. The project was advertised on social media and the research team approached friends and family members; the link was shared among those who accepted to participate through social media applications (WhatsApp, Instagram, Facebook). Consequently, participants were asked to forward the link to other friends and family members they know, explaining the snowball technique. Inclusion criteria for participation included being of a resident and citizen of Lebanon, aged over 18 years, and willing to participate (Further details about data collection can be found in [102]). After providing digital informed consent, participants were asked to complete the instruments described above, which were presented in a pre-randomized order to control for order effects.

### Participants

A total of 570 Lebanese adults enrolled in this study, with a mean age of 24.59 years (*SD* = 6.75) and 68.6% women. Other sample characteristics are displayed in Table 1.

**Table 1:** Sociodemographic characteristics of the participants

Variable	Total sample (*N* = 570)	First split-half subsample (*n* = 277)	Second split-half subsample *(n* = 293)
Gender			
Men	179 (31.4%)	90 (32.5%)	89 (30.4%)
Women	391 (68.6%)	187 (67.5%)	204 (69.6%)
Marital status			
Single	477 (83.7%)	232 (83.8%)	245 (83.6%)
Married	93 (16.3%)	45 (16.2%)	48 (16.4%)
Education			
Secondary or less	29 (5.1%)	18 (6.5%)	11 (3.8%)
University	541 (94.9%)	259 (93.5%)	282 (96.2%)
Region of living			
Urban	280 (49.1%)	141 (50.9%)	139 (47.4%)
Rural	290 (50.9%)	136 (49.1%)	154 (52.6%)
	**Mean ± SD**		
Age (in years)	24.59 ± 6.75	24.88 ± 7.76	24.32 ± 5.63
Household crowding index (person/room)	1.10 ± 0.51	1.13 ± 0.56	1.07 ± 0.45

### Measures

#### Adaptation and development of the SSS-CD

As mentioned above, choosing to develop a scale assessing social stigma attached to chronic diseases and based on items derived from Green’s scale [68] was mainly guided by the theoretical viewpoints that social stigma shares substantial similarities across a broad range of chronic diseases [41]. Regarding the scale adaptation and development procedure, the initial version of all 15 items of the original scale was examined for relevance by a panel composed of specialist clinicians and researchers with expertise in the field of stigma. The review involved the following two aspects: (a) relevance of the original 15 items to the scale (more particularly, to the targeted construct and population), and (b) suitability for the Arab cultural background. As previously mentioned, the main modification consisted in changing the word “HIV” in each item of the initial version of the scale to “chronic diseases”. After reviewing the initial pool of 15 items, two items were considered as being too specific to HIV as a sexually transmitted disease, and were thus removed (i.e., dirtiness and having a child). Indeed, in Arab countries in particular, homosexuality and non-marital sex are prohibited; therefore, HIV is viewed as “a moral disease” and people with HIV are considered as deviant for having violated socio-religious rules and moral commitments [103]. This might lead to perceptions of dirtiness and inability or inability/non-deservedness of having children. One item was judged as too narrow and very specific to people with HIV (i.e. “Prisoners with chronic diseases should be segregated”). As such this item has been reworded as follows: “People with chronic diseases should be segregated in the workplace”, which also enables to include the concept of stigma from co-workers, colleagues or employers in the workplace that was missing in the scale. As such, the analyses were performed on 13 selected items out of the 15 initial items. These items are scored from 1 (strongly disagree) to 4 (strongly agree), with higher scores indicating more stigmatized attitudes toward people living with chronic diseases.

#### Translation procedure

This translation process was done in conformity to international guidelines required for a scale’s validation [104, 105]. The cross-cultural adaptation was initiated by a two-step translation procedure: a forward translation (from English to Arabic), then a backward translation (from Arabic to English), performed by two distinct healthcare professionals, native Arabic speakers and fluent in English. The initial and translated English versions were compared to detect and later eliminate any inconsistencies; the procedure was repeated until all the inconsistencies were solved. At the end, the final Arabic version was approved by a committee constituted of two psychologists and two psychiatrists. A pilot test was done on 20 participants to ensure that all questions were well understood. No changes were done afterwards.

### Other measures

*The* Standardized Stigmatization Questionnaire (SSQ) [101] assesses the perception of social stigmatization and the predisposition to enact stigmatization through three dimensions: Social self-interest (e.g., “Would most people avoid talking to him if possible?”), Evolutionary self-interest (e.g., “Would most people be happy if he were to work together with them in their workplace?”), and Psychological self-interest (e.g., “Do most people think one of the main causes of his condition is a lack of moral strength or will power?”). The SSQ is a 13-item psychometric instrument used with a 4-point Likert scale. Higher scores indicate higher stigmatization (ω = 0.83).

*Demographics.* Participants were asked to provide their demographic details consisting of age, gender, highest educational attainment, region of living, marital status. The number of persons and rooms in the house were used to compute the household crowding index (person/room); the higher the number, the lower the socioeconomic status [106].

#### Analytic Strategy

**Data treatment.** There were no missing responses in the dataset. To examine the factor structure of the SSS-CD, we used an EFA-to-CFA strategy [107]. To ensure adequate sample sizes for both EFA and CFA, we split the main sample using an SPSS computer-generated random technique; sample characteristics of the two split-halves are reported in Table 1. There were no significant differences between the two subsamples in terms of mean age, *t*(568) = 0.984, *p* = .326, education level χ^2^(1) = 2.22, *p* = .136, marital status χ^2^(1) = 0.002, *p* = .965 and the distribution of women and men, χ^2^(1) = 0.296, *p* = .587.

**Exploratory factor analysis.** To explore the factor structure of SSS-CD, we computed a principal-axis EFA with the first split-half subsample using the FACTOR software [108, 109]. A minimum sample of 120 participants was calculated according to Comree and Lee [110], who suggested 10 participants per scale’s item. We verified all requirements related to item-communality [111], average item correlations, and item-total correlations [112]. The Kaiser-Meyer-Olkin (KMO) measure of sampling adequacy (which should ideally be ≥ 0.80) and Bartlett’s test of sphericity (which should be significant) ensured the adequacy of our sample [113]. The procedure for determining the number of factors to extract was parallel analysis [114] using the Pearson correlation matrix. Weighted Root Mean Square Residual (WRMR) was also calculated to assess the model fit (values < 1 have been recommended to represent good fit; [115]. Item retention was based on the recommendation that items with “fair” loadings and above (i.e., ≥ 0.4) [116].

**Confirmatory factor analysis.** We used data from the second split-half to conduct a CFA using the SPSS AMOS v.29 software. A previous study suggested that the minimum sample size to conduct a confirmatory factor analysis ranges from 3 to 20 times the number of the scale’s variables [117], which was surpassed in our study. Our intention was to test the model extracted from the EFA. Parameter estimates were obtained using the maximum likelihood method and fit indices. The normed model chi-square (χ²/df), the Steiger-Lind root mean square error of approximation (RMSEA), the Tucker-Lewis Index (TLI) and the comparative fit index (CFI). Values ≤ 3 for χ²/df, and ≤ 0.08 for RMSEA, and 0.90 for CFI and TLI indicate acceptable fit of the model to the data [118].

**Gender invariance.** To examine gender invariance of IEQ scores, we conducted multi-group CFA [119] using the second split-half subsample. Measurement invariance was assessed at the configural, metric, and scalar levels [120]. Configural invariance implies that the latent IEQ variable(s) and the pattern of loadings of the latent variable(s) on indicators are similar across gender (i.e., the unconstrained latent model should fit the data well in both groups). Metric invariance implies that the magnitude of the loadings is similar across gender; this is tested by comparing two nested models consisting of a baseline model and an invariance model. Lastly, scalar invariance implies that both the item loadings and item intercepts are similar across gender and is examined using the same nested-model comparison strategy as with metric invariance [119]. Following previous recommendations [119, 121], we accepted ΔCFI ≤ 0.010 and ΔRMSEA ≤ 0.015 or ΔSRMR ≤ 0.010 (0.030 for factorial invariance) as evidence of invariance. We aimed to test for gender differences on latent IEQ scores using an independent-samples *t*-test only if scalar or partial scalar invariance were established.

**Further analyses.** Composite reliability in both subsamples was assessed using McDonald’s ω and its associated 95% CI, with values greater than 0.70 reflecting adequate composite reliability [122]. McDonald’s ω was selected as a measure of composite reliability because of known problems with the use of Cronbach’s α (e.g., [123]. To assess convergent and criterion-related validity, we examined bivariate correlations between SSS-CD and SSQ scores using the total sample. All scores had normal distribution, as identified by skewness and kurtosis values varying between − 1 and + 1 [124]; therefore, Pearson correlation test was used. Based on Cohen’s recommendations [125], values ≤ 0.10 were considered weak, ~ 0.30 were considered moderate, and ~ 0.50 were considered strong correlations.

## Results

### Exploratory factor analysis of the SSS-CD

*Factor analysis on sample 1.* The Bartlett’s test of sphericity, χ^2^(36) = 678.8, *p* < .001, and KMO (0.805) indicated that the SSS-CD items had adequate common variance for factor analysis. The results of the EFA revealed one factor, which explained 55.69% of the common variance. Items 6 and 7 were removed because of low communality (< 0.3). The WRMR value was also adequate (= 0.091; 95% CI 0.082-0.097), indicating good fit of the model.

*Factor structure congruence and composite reliability.* The factor loadings reported in Table 2. McDonald’s ω was adequate in women (ω = 0.67), men (ω = 0.76), and the total subsample (ω = 0.72).

### Confirmatory Factor Analysis of the ***SSS-CD***

CFA indicated that fit indices of the 2-factor model of the SSS-CD were acceptable: χ^2^/df = 115.52/43 = 2.69, RMSEA = 0.076 (90% CI 0.059, 0.093), SRMR = 0.070, CFI = 0.928, TLI = 0.908. However, item 11 had a low factor loading (= 0.32). We removed it and re-did the analysis; the fit indices improved as follows: χ^2^/df = 92.95/34 = 2.73, RMSEA = 0.077 (90% CI 0.059, 0.096), SRMR = 0.062, CFI = 0.939, TLI = 0.919. The standardized estimates of factor loadings were all adequate (see Table 2).

### Composite reliability

Composite reliability of scores was adequate in women (ω = 0.71), men (ω = 0.72), and the total sample (ω = 0.71).

**Table 2:** The 10 items of the Social Stigma Scale of Chronic Diseases (SSS-CD) in English and Factor Loadings Derived from the Exploratory Factor Analyses (EFA) in the First Split-Half Subsample, and Standardized Estimates of Factor Loadings from the Confirmatory Factor Analysis (CFA) in the Second Split-Half Subsample

	EFA		CFA
Item	Factor 1	Factor 2	
1. People with chronic diseases are as intelligent as anybody else*		0.53	0.75
2. People with chronic diseases are not to be trusted	0.69		0.66
3. Being with chronic diseases says nothing about who you are*		0.62	0.43
4. People with chronic diseases are no different from anybody else*		0.68	0.77
5. People with chronic diseases should be ashamed on themselves	0.76		0.80
6. People with chronic diseases have nothing to feel guilty about*		0.75	0.42
7. You become with chronic diseases by being weak-willed or foolish	0.77		0.80
8. People with chronic diseases should be segregated in the workplace	0.76		0.74
9. It is safe for people with chronic diseases to work with children*		0.70	0.56
10. People with chronic diseases must expect some restrictions on their freedom*	0.69		0.72

*refers to reversed scoring items; Factor 1 = Negative attitude towards stigma; Factor 2 = Positive attitude towards stigma

### Gender invariance of the SSS-CD

As reported in Table 3, indices suggested that configural, metric, and scalar invariance was supported across gender. In terms of stigma towards patients, men (*M* = 19.48, *SD* = 5.78) had a higher mean STP score compared to women (*M* = 18.10, *SD* = 5.24) in the second split-half subsample, *t*(291) = 2.015, *p* = .045, d = 0.250.

**Table 3:** Measurement Invariance of the Social Stigma Scale of Chronic Diseases (SSS-CD) items Across Gender in the Second Split-Half Subsample

Model	χ²	*df*	CFI	RMSEA	SRMR	Model Comparison	Δχ²	ΔCFI	ΔRMSEA	ΔSRMR	Δ*df*	*p*
Configural	131.02	68	0.935	0.056	0.064							
Metric	143.33	76	0.931	0.055	0.083	Configural vs. metric	12.31	0.004	0.001	0.019	8	0.138
Scalar	146.55	84	0.936	0.051	0.083	Metric vs. scalar	3.22	0.005	0.004	< 0.001	8	0.919

*Note.* CFI = Comparative fit index; RMSEA = Steiger-Lind root mean square error of approximation; SRMR = Standardised root mean square residual

### Convergent validity

Higher SSS-CD scores were moderately-to-strongly significantly associated with more social self-interest, evolutionary self-interest, and psychological self-interest (Table 4).

**Table 4:** Correlations of the Social Stigma Scale of Chronic Diseases (SSS-CD) scores with the other measures in the total sample

	1	2	3	4	5
1. Social stigma of chronic diseases (SSS-CD)	1				
2. Social self-interest	0.40***	1			
3. Evolutionary self-interest	0.37***	0.61***	1		
4. Psychological self-interest	0.42***	0.48***	0.28***	1	
5. Age	0.05	0.09*	0.07	0.04	1

*p < .05; ** *p* < .01; *** *p* < .001

## Discussion

A growing body of literature has demonstrated that public stigma is commonly experienced by people living with chronic illnesses [126, 127], in Arab countries and globally. However, there is a lack of psychometrically sound instruments evaluating social stigma across chronic illnesses [36, 41]. As a contribution to this literature, we sought to adapt and develop a measure to assess public attitudes towards people living with chronic diseases in Arabic-speaking populations, the 10-item Social Stigma Scale of Chronic Diseases (SSS-CD). Our findings suggest that the SSS-CD has robust psychometric qualities, with adequate factorial and convergent validity, as well as gender invariance. Strong evidence for good internal consistency was also demonstrated by a McDonald’s omega value of 0.73 for the total score. Using McDonald’s Omega is recommended for examining reliability since it gives a more optimal estimation measure of composite reliability compared to Cronbach Alpha [128], thus representing a strength of our analysis. The reliability of a measure, as evaluated using its internal consistency, is a necessary step during the validation process. As measurement error can be present in content sampling, internal consistency is considered as the consistency of the survey results [129]. Internal consistency allows to ensure that each item on the SSS-CD reflects the social stigma construct it is intended to assess. In other words, this psychometric property indicates to what extent the 10 items of the SSS-CD are inter-correlated, and whether they are consistent in measuring the same construct [129].

In terms of the factorial validity of the SSS-CD, our results are consistent with previous work showing that scores are unidimensional. Indeed, in the original validation study, Green [68] showed that that the initially theoretically driven three separate domains (cognitive features, victim blaming, and treatment from society) did not operate independently in a sample of Scottish community adults. Indeed, fit of the unidimensional model of SSS-CD scores in the present study was adequate when tested using both EFA and CFA, with the latter achieving adequate fit without the need for modifications. One broad conclusion that may preliminarily be drawn on the basis of these results is that the SSS-CD retains a unidimensional structure across a wide range of chronic diseases, which supports that the one-factor model of social stigma surrounding chronic conditions holds up cross culturally. The unidimensional structure of the SSS-CD is advantageous, as it allows to easily calculate a total score. This may foster its application in large screening studies and facilitate cross-national comparisons of scores. We are aware, however, that to confirm our assumptions it will be important to examine the extent to which scores on the SSS-CD are invariant across different diseases in future research.

Our results also indicated that the unidimensional factor structure of SSS-CD scores was not identical across women and men (in our EFA) and achieved full scalar variance (in our CFA) where stigma was higher among men compared to women consistent with other studies [130, 131]. For instance, Chinese American women tended to endorse that health insurance policies should provide coverage for the dementia-related care needs more than did their male counterpart [129]. This finding can be explained by the fact that women held positive attitudes and are more likely to seek help for health issues than men [132]. Men may seek counseling less often than women and men may internalize public stigma more strongly than women [131]. This may be explained by the fact that traditional gender roles lead society to actually stigmatize men with disease diagnosis to a greater degree than women [130]. In contrast with these findings, other studies documented greater stigma levels towards chronic illnesses such as cancer [133], HIV [134], and human papillomavirus (HPV) infection [135] in women from Kenya, USA and Senegal as compared to men. Even though these discrepancies may be explained by multiple determinants, such as socio-cultural factors and gender roles, future studies still need to explore gender influences in chronic illnesses social stigma in different contexts and backgrounds. In addition, it is crucial that gender invariance be established and taken into account in the design and validation of chronic illness stigma measures, to ensure accurate gender comparisons. Finally, our findings revealed that all three dimensions of stigmatization (social, psychological and evolutionary stigmatization) as assessed using the SSQ were positively correlated with SSS-CD scores. The SSQ was designed to measure stigma held by the general public towards people with chronic illness [101]. The SSQ is divided into three components of stigmatization, i.e. social self-interest which reflects social distance of the respondent to the chronically ill, evolutionary self-interest which assesses the way how the respondent is likely to react, and psychological self-interest, which describes the allocation of negative attributes (e.g., ‘badness’ and ‘failure’) to the chronically ill in order to provide the self with relative self-glorification. According to Bagozzi [136] convergent validity refers to the fact that “measures of the same construct should be highly intercorrelated among themselves and uniform in the pattern of intercorrelations”. Given that the SSQ and the SSS-CD both capture similar information on social stigma, positive correlations between the total SSS-CD scores and all three subscores of the SSQ provide evidence for the relatively strong convergent validity, which, in turn, is an indication of construct validity. This finding implies that the SSS-CD is a valid self-report tool, and underscores its utility of in measuring social stigma towards the chronically ill among Arabic-speaking community adults.

### Limitations

A number of limitations of the present study could be improved in future studies. First, the method of recruitment was performed only among a Lebanese sample of community people, which might compromise the generalizability of our conclusions to Arabic-speaking adults from other Arab and non-Arab countries. We are not capable of retrieving the response rate from Google forms, which predisposes us to a selection bias. In addition, we adopted a web-based method for data collection, which mostly attracted women (68.6%) and highly educated (94.9%) participants. This may limit the generalizability of our findings. This may be particularly important given that the vast majority of participants are educated and females. Larger and cross-national validation studies is still need to confirm the robustness of the scale across Arab countries. A further limitation of the present work was that the SSQ scale is not validated in Arabic and we did not assess other psychometric properties such as divergent validity or test-retest reliability, which should be rectified in future research.

## Conclusion

The present study supports the adequate psychometric properties of the 10-item Social Stigma Scale of Chronic Diseases (SSS-CD) in terms of factor structure, reliability, gender invariance, and convergent validity. We thus preliminarily recommend its use among Arabic-speaking people from the general population to measure public attitudes towards people living with chronic diseases. Providing this simple, convenient and reliable measure will hopefully enable to draw a clear overview of the prevalence and characteristics of social stigma attached to chronic diseases in Arabic-speaking communities. This would, in turn, assist decision makers and stakeholders in implementing effective and culturally tailored anti-stigma strategies aiming at improving community integration of people living with chronic diseases in Arab settings. However, given that this was the first study to examine the psychometric properties of the SSS-CD, the present findings and conclusions should be considered tentative pending future cross-national validation studies. Also, additional studies attempting to address the above-mentioned limitations are warranted.

## Data Availability

All data generated or analyzed during this study are not publicly available but are available upon a reasonable request from the corresponding author (S.H.).

## References

[1] Kaneda T, El-Saharty S (2017). Curbing the noncommunicable disease epidemic in the Middle East and North Africa: Prevention among young people is the key, Population Reference Bureau.

[2] Rahim HFA, Sibai A, Khader Y, Hwalla N, Fadhil I, Alsiyabi H, Mataria A, Mendis S, Mokdad AH, Husseini A (2014). Non-communicable diseases in the arab world. The Lancet.

[3] WHO. Global Health estimates 2020: deaths by cause, Age, Sex, by Country and by Region, 2000–2019. In.: World Health Organization Geneva; 2020.

[4] Bashir M, Alshamsi M, Almahrooqi S, Alyammahi T, Alhammadi W, Alshehhi S, Alhosani H, Alhammadi F, Al-Maskari F (2022). Prevalence of chronic diseases among United Arab Emirates University students: cross-sectional study. Eur J Pub Health.

[5] Organization WH. Noncommunicable diseases progress monitor 2020 [Internet]. Genebra: World Health Organization; 2020 In.

[6] Safiri S, Noori M, Nejadghaderi SA, Mousavi SE, Sullman MJ, Araj-Khodaei M, Singh K, Kolahi A-A, Gharagozli K (2023). The burden of Parkinson’s disease in the Middle East and North Africa region, 1990–2019: results from the global burden of disease study 2019. BMC Public Health.

[7] Casati J, Toner BB, De Rooy EC, Drossman DA, Maunder RG (2000). Concerns of patients with inflammatory bowel disease. Dig Dis Sci.

[8] Kellett S, Gilbert P (2001). Acne: a biopsychosocial and evolutionary perspective with a focus on shame. Br J Health Psychol.

[9] Dolezal L, Lyons B (2017). Health-related shame: an affective determinant of health?. Med Humanit.

[10] Earnshaw VA, Quinn DM, Park CL (2012). Anticipated stigma and quality of life among people living with chronic illnesses. Chronic Illn.

[11] Goffman E. Stigma: notes on the Management of Spoiled Identity (kindle edition). *Touchstone* 1963.

[12] Karidi MV, Vassilopoulou D, Savvidou E, Vitoratou S, Maillis A, Rabavilas A, Stefanis CN (2015). Bipolar disorder and self-stigma: a comparison with schizophrenia. J Affect Disord.

[13] King M, Dinos S, Shaw J, Watson R, Stevens S, Passetti F, Weich S, Serfaty M (2007). The Stigma Scale: development of a standardised measure of the stigma of mental illness. Br J Psychiatry.

[14] Goffman E. Stigma: notes on the management of spoiled identity. Simon and schuster; 2009.

[15] Rewerska-Juśko M, Rejdak K (2020). Social Stigma of People with Dementia. J Alzheimers Dis.

[16] Jackowska E (2009). [Stigma and discrimination towards people with schizophrenia–a survey of studies and psychological mechanisms]. Psychiatr Pol.

[17] Chapple A, Ziebland S, McPherson A (2004). Stigma, shame, and blame experienced by patients with lung cancer: qualitative study. BMJ.

[18] Butt G (2008). Stigma in the context of hepatitis C: concept analysis. J Adv Nurs.

[19] Møller V, Erstad I (2007). Stigma associated with tuberculosis in a time of HIV/AIDS: narratives from the Eastern Cape, South Africa. South Afr Rev Sociol.

[20] Person B, Bartholomew LK, Gyapong M, Addiss DG, van den Borne B (2009). Health-related stigma among women with lymphatic filariasis from the Dominican Republic and Ghana. Soc Sci Med.

[21] Mak WW, Cheung RY, Law RW, Woo J, Li PC, Chung RW (2007). Examining attribution model of self-stigma on social support and psychological well-being among people with HIV+/AIDS. Soc Sci Med.

[22] Shiu AT-Y, Kwan JJY-M, Wong RY-M (2003). Social stigma as a barrier to diabetes self-management: implications for multi-level interventions. J Clin Nurs (Print).

[23] Sleeth C, Drake K, Labiner DM, Chong J (2016). Felt and enacted stigma in elderly persons with epilepsy: a qualitative approach. Epilepsy Behav.

[24] Huang J, Guan ML, Balch J, Wu E, Rao H, Lin A, Wei L, Lok AS (2016). Survey of hepatitis B knowledge and stigma among chronically infected patients and uninfected persons in Beijing, China. Liver Int.

[25] Earnshaw VA, Kalichman SC. Stigma experienced by people living with HIV/AIDS. Stigma, discrimination and living with HIV/AIDS. edn.: Springer; 2013: 23–38.

[26] Vaughn-Sandler V, Sherman C, Aronsohn A, Volk ML (2014). Consequences of perceived stigma among patients with cirrhosis. Dig Dis Sci.

[27] Markowitz S, Friedman MA, Arent SM (2008). Understanding the relation between obesity and depression: causal mechanisms and implications for treatment. Clin Psychol Sci Pract.

[28] West MD, Dye AN, McMahon BT (2006). Epilepsy and workplace discrimination: population characteristics and trends. Epilepsy Behav.

[29] Sayles JN, Ryan GW, Silver JS, Sarkisian CA, Cunningham WE (2007). Experiences of social stigma and implications for healthcare among a diverse population of HIV positive adults. J Urban Health.

[30] Leventhal H, Halm E, Horowitz C, Leventhal EA, Ozakinci G. Living with chronic illness: a contextualized, self-regulation approach. Sage Handb health Psychol 2004:197–240.

[31] Kurzban R, Leary MR (2001). Evolutionary origins of stigmatization: the functions of social exclusion. Psychol Bull.

[32] Earnshaw VA, Quinn DM, Kalichman SC, Park CL (2013). Development and psychometric evaluation of the chronic illness anticipated stigma scale. J Behav Med.

[33] Weiler DM, Crist JD (2009). Diabetes self-management in a latino social environment. Diabetes Educ.

[34] Butt G, Paterson BL, McGuinness LK (2008). Living with the stigma of hepatitis C. West J Nurs Res.

[35] Baker GA, Brooks J, Buck D, Jacoby A (2000). The stigma of epilepsy: a european perspective. Epilepsia.

[36] Schabert J, Browne JL, Mosely K, Speight J (2013). Social stigma in diabetes. The Patient-Patient-Centered Outcomes Research.

[37] McMaugh A (2011). En/countering disablement in school life in Australia: children talk about peer relations and living with illness and disability. Disabil Soc.

[38] Holzemer WL, Human S, Arudo J, Rosa ME, Hamilton MJ, Corless I, Robinson L, Nicholas PK, Wantland DJ, Moezzi S (2009). Exploring HIV stigma and quality of life for persons living with HIV infection. J Assoc Nurses AIDS Care.

[39] Earnshaw VA, Quinn DM (2012). The impact of stigma in healthcare on people living with chronic illnesses. J Health Psychol.

[40] Link BG, Phelan JC (2006). Stigma and its public health implications. The Lancet.

[41] Van Brakel WH (2006). Measuring health-related stigma–a literature review. Psychol Health Med.

[42] Soliman MM (2020). Feeling of stigmatization and satisfaction with life among arabic psoriatic patients. Saudi Pharm J.

[43] Dimitrov D, Matusiak Ł, Szepietowski J (2019). Stigmatization in arabic psoriatic patients in the United Arab Emirates–a cross sectional study. Adv Dermatology Allergology/Postępy Dermatologii i Alergologii.

[44] Al-Zamel LA, Al-Thunayan SF, Al-Rasheed AA, Alkathiri MA, Alamri F, Alqahtani F, Alali AS, Almohammed OA, Asiri YA, Bashatah AS (2021). Validation and cultural adaptation of explanatory model interview catalogue (EMIC) in assessing stigma among recovered patients with COVID-19 in Saudi Arabia. Int J Environ Res Public Health.

[45] Alhatemi NAY, Hassanen RH, Alnawafleh KA (2018). The perceptions of tuberculosis and its Stigma among TB Patients in Damascus, Syria Arab Republic. Public Health and Preventive Medicine.

[46] Tayeb HO (2019). Epilepsy stigma in Saudi Arabia: the roles of mind–body dualism, supernatural beliefs, and religiosity. Epilepsy Behav.

[47] Karimi N, Akbarian SA. Knowledge and attitude toward epilepsy of close family members of people with epilepsy in North of Iran. *Advances in Medicine* 2016, 2016. 10.1155/2016/8672853 PMC5220387 28116347

[48] Cohen M, Werner P, Azaiza F (2009). Emotional reactions of arab lay persons to a person with Alzheimer’s disease. Aging and Mental Health.

[49] Soffer M (2020). HIV/AIDS-related beliefs among israeli arab‐palestinian women. Health Soc Care Commun.

[50] Rao D, Choi SW, Victorson D, Bode R, Peterman A, Heinemann A, Cella D (2009). Measuring stigma across neurological conditions: the development of the stigma scale for chronic illness (SSCI). Qual Life Res.

[51] Elgohari HM, Bassiony MM, Sehlo MG, Youssef UM, Ali HM, Shahin I, Elrafey DS, Mahdy RS (2021). COVID-19 infection stigma scale: psychometric properties. Egypt J Neurol Psychiatry Neurosurg.

[52] Maffoni M, Giardini A, Pierobon A, Ferrazzoli D, Frazzitta G. Stigma experienced by Parkinson’s disease patients: a descriptive review of qualitative studies. *Parkinson’s Disease* 2017, 2017. 10.1155/2017/7203259 PMC5294385 28243481

[53] Ghorbanibirgani A, Fallahi-Khoshknab M, Zarea K, Abedi H. The lived experience of psoriasis patients from social stigma and rejection: a qualitative study. Iran Red Crescent Med J 2016, 18(7). 10.5812/ircmj.27893 PMC5026766 27656290

[54] Harris M, Guy D, Picchio CA, White TM, Rhodes T, Lazarus JV (2021). Conceptualising hepatitis C stigma: a thematic synthesis of qualitative research. Int J Drug Policy.

[55] Hasan Shiri F, Mohtashami J, Manoochehri H, Rohani C (2022). Cancer Stigma and its Consequences and influencing factors in iranian society: a qualitative study. J Qualitative Res Health Sci.

[56] Fernandes PT, Salgado PC, Noronha ALA, Barbosa FD, Souza EA, Sander JW, Li LM (2007). Prejudice towards chronic diseases: comparison among epilepsy, AIDS and diabetes. Seizure.

[57] Kim S, Eccleston C, Klekociuk S, Cook PS, Doherty K. Development and psychometric evaluation of the Dementia Public Stigma Scale. Int J Geriatr Psychiatry 2022, 37(2). 10.1002/gps.5672 34997624

[58] Nochaiwong S, Ruengorn C, Awiphan R, Kanjanarat P, Ruanta Y, Phosuya C, Boonchieng W, Nanta S, Chongruksut W, Thavorn K (2021). COVID-19 Public Stigma Scale (COVID-PSS): development, validation, psychometric analysis and interpretation. BMJ Open.

[59] Fernandes PT, Salgado PC, Noronha AL, Sander JW, Li LM (2007). Stigma scale of Epilepsy: validation process. Arq Neuropsiquiatr.

[60] Lillis J, Luoma JB, Levin ME, Hayes SC (2010). Measuring weight self-stigma: the weight self‐stigma questionnaire. Obesity.

[61] odibo MW, Idemudia ES, Olasupo MO (2018). Stigma and chronic illness: a comparative study of people living with HIV and/or AIDS and people living with hypertension in Limpopo Province, South Africa. Curationis.

[62] Nyblade LC (2006). Measuring HIV stigma: existing knowledge and gaps. Psychol Health Med.

[63] Yang LH, Kleinman A, Link BG, Phelan JC, Lee S, Good B (2007). Culture and stigma: adding moral experience to stigma theory. Soc Sci Med.

[64] Hatzenbuehler ML, Link BG. Introduction to the special issue on structural stigma and health. Social Science & Medicine; 2014. 10.1016/j.socscimed.2013.12.017 24445152

[65] Hazazi A, Wilson A (2022). Noncommunicable diseases and health system responses in Saudi Arabia: focus on policies and strategies. A qualitative study. Health Res Policy Syst.

[66] Alenzi EO, Fatima W, Amara A, Imran M, Shah SSH, Elbilgahy AA, Fawzy MS, Abu-Negm LM, Mujtaba MA, Jacinto-Caspillo I. A systematic review of chronic Diseases and their prevalence among the Population of Northern Borders Province (NBP) in Saudi Arabia. J Multidisciplinary Healthc 2023:1047–56. 10.2147/JMDH.S401001 PMC10120835 37089278

[67] Stangl AL, Lilleston P, Mathema H, Pliakas T, Krishnaratne S, Sievwright K, Bell-Mandla N, Vermaak R, Mainga T, Steinhaus M (2019). Development of parallel measures to assess HIV stigma and discrimination among people living with HIV, community members and health workers in the HPTN 071 (PopART) trial in Zambia and South Africa. J Int AIDS Soc.

[68] Green G (1995). Attitudes towards people with HIV: are they as stigmatizing as people with HIV perceive them to be?. Soc Sci Med.

[69] Fernandes PT, Salgado PCB, Noronha ALA, Barbosa FD, Souza EAP, Sander JW, Li LM (2007). Prejudice towards chronic diseases: comparison among epilepsy, AIDS and diabetes. Seizure.

[70] Bloom JR, Kessler L. Emotional support following cancer: a test of the stigma and social activity hypotheses. J Health Soc Behav 1994:118–33. 8064120

[71] Herbert TB, Dunkel-Schetter C. Negative social reactions to victims: An overview of responses and their determinants. 1992.

[72] Saki M, Mohammad Khan Kermanshahi S, Mohammadi E, Mohraz M (2015). Perception of patients with HIV/AIDS from stigma and discrimination. Iran Red Crescent Med J.

[73] Fife BL, Wright ER (2000). The dimensionality of stigma: a comparison of its impact on the self of persons with HIV/AIDS and cancer. J Health Soc Behav.

[74] Browne JL, Ventura A, Mosely K, Speight J (2014). I’m not a druggie, I’m just a diabetic’: a qualitative study of stigma from the perspective of adults with type 1 diabetes. BMJ open.

[75] Mayor R, Gunn S, Reuber M, Simpson J (2022). Experiences of stigma in people with epilepsy: a meta-synthesis of qualitative evidence. Seizure.

[76] Dodor EA, Neal K, Kelly S (2008). An exploration of the causes of tuberculosis stigma in an urban district in Ghana. Int J Tuberc Lung Dis.

[77] Parker M, Barlow S, Hoe J, Aitken LM (2022). The bubble of normalisation: a qualitative study of carers of people with dementia who do not seek help for a diagnosis. J Geriatr Psychiatr Neurol.

[78] Mak WW, Mo PK, Cheung RY, Woo J, Cheung FM, Lee D (2006). Comparative stigma of HIV/AIDS, SARS, and tuberculosis in Hong Kong. Soc Sci Med.

[79] Forhan M, Salas XR (2013). Inequities in healthcare: a review of bias and discrimination in obesity treatment. Can J diabetes.

[80] Grodensky CA, Golin CE, Jones C, Mamo M, Dennis AC, Abernethy MG, Patterson KB (2015). I should know better”: the roles of relationships, spirituality, disclosure, stigma, and shame for older women living with HIV seeking support in the South. J Assoc Nurses AIDS Care.

[81] Craig G, Daftary A, Engel N, O’driscoll S, Ioannaki A (2017). Tuberculosis stigma as a social determinant of health: a systematic mapping review of research in low incidence countries. Int J Infect Dis.

[82] Rice DR, Cisse FA, Hamani ABD, Tassiou NR, Sakadi F, Bah AK, Othon GC, Conde ML, Diawara K, Traoré M (2021). Epilepsy stigma in the Republic of Guinea and its socioeconomic and clinical associations: a cross-sectional analysis. Epilepsy Res.

[83] Browne JL, Ventura AD, Mosely K, Speight J (2016). Measuring the stigma surrounding type 2 diabetes: development and validation of the type 2 diabetes Stigma Assessment Scale (DSAS-2). Diabetes Care.

[84] Inagaki S, Matsuda T, Muramae N, Abe K, Kato K (2022). Diabetes-related shame among people with type 2 diabetes: an internet-based cross-sectional study. BMJ Open Diabetes Research and Care.

[85] Else-Quest NM, LoConte NK, Schiller JH, Hyde JS (2009). Perceived stigma, self-blame, and adjustment among lung, breast and prostate cancer patients. Psychol Health.

[86] Batsis JA, Zagaria AB, Brooks E, Clark MM, Phelan S, Lopez-Jimenez F, Bartels SJ, Rotenberg S, Carpenter-Song E (2021). The use and meaning of the term obesity in rural older adults: a qualitative study. J Appl Gerontol.

[87] Jankowiak B, Kowalewska B, Krajewska-Kułak E, Kowalczuk K, Khvorik DF (2021). The sense of stigmatization in patients with Plaque Psoriasis. Dermatology.

[88] Kalichman SC, Simbayi LC, Cloete A, Mthembu PP, Mkhonta RN, Ginindza T (2009). Measuring AIDS stigmas in people living with HIV/AIDS: the internalized AIDS-Related Stigma Scale. AIDS Care.

[89] Buchman DZ, Ho A, Illes J (2016). You present like a drug addict: patient and clinician perspectives on trust and trustworthiness in chronic pain management. Pain Med.

[90] Joachim G, Acorn S (2003). Life with a rare chronic disease: the scleroderma experience. J Adv Nurs.

[91] Robson C, Lian OS. “Blaming, shaming, humiliation”: stigmatising medical interactions among people with non-epileptic seizures. Wellcome open research 2017, 2. 10.12688/wellcomeopenres.12133.2 PMC5664997 29152594

[92] Li J, Assanangkornchai S, Lu L, Jia M, McNeil EB, You J, Chongsuvivatwong V (2016). Development of internalized and personal stigma among patients with and without HIV infection and occupational stigma among health care providers in Southern China. Patient Prefer Adherence.

[93] Jacoby A, Gorry J, Gamble C, Baker GA (2004). Public knowledge, private grief: a study of public attitudes to epilepsy in the United Kingdom and implications for stigma. Epilepsia.

[94] Shen MJ, Wellman JD (2019). Evidence of palliative care stigma: the role of negative stereotypes in preventing willingness to use palliative care. Palliat Support Care.

[95] Beverly EA, Guseman EH, Jensen LL, Fredricks TR (2019). Reducing the stigma of diabetes in medical education: a contact-based educational approach. Clin Diabetes.

[96] Flint SW. Time to end weight stigma in healthcare. EClinicalMedicine 2021, 34. 10.1016/j.eclinm.2021.100810 PMC8042345 33870153

[97] Gengler AM (2014). Not tonight: migraine and the politics of gender and health.

[98] Pope CK, Shoultz G (2012). An interdisciplinary approach to HIV/AIDS stigma and discrimination in Belize: the roles of geography and ethnicity. GeoJournal.

[99] Børte S, Ottersen VS. Stigmatization of children with chronic diseases, exemplified by type 1 diabetes mellitus. Differences between India and Norway; 2012.

[100] Sibulwa S, Chansa-Kabali T, Hapunda G (2019). Every part of me has changed”—shared lived experiences of adolescents living with cancer in Zambia. Health Psychol Open.

[101] Haghighat R (2005). The development of an instrument to measure stigmatization: factor analysis and origin of stigmatization. Eur J psychiatry.

[102] Fekih-Romdhane F, Malaeb D, Dabbous M, Hallit R, Obeid S, Hallit S (2023). Psychometric properties of an arabic translation of the external and internal shame scale (EISS). BMC Psychiatry.

[103] Badahdah AM (2010). Stigmatization of persons with HIV/AIDS in Saudi Arabia. J Transcult Nurs.

[104] Beaton D, Bombardier C, Guillemin F, Ferraz MB (2002). Recommendations for the cross-cultural adaptation of health status measures. New York: American Academy of Orthopaedic Surgeons.

[105] Beaton DE, Bombardier C, Guillemin F, Ferraz MB (2000). Guidelines for the process of cross-cultural adaptation of self-report measures. Spine.

[106] Melki I, Beydoun H, Khogali M, Tamim H, Yunis K (2004). Household crowding index: a correlate of socioeconomic status and inter-pregnancy spacing in an urban setting. J Epidemiol Community Health.

[107] Swami V, Barron D (2019). Translation and validation of body image instruments: Challenges, good practice guidelines, and reporting recommendations for test adaptation. Body image.

[108] Lorenzo-Seva U, Ten Berge J (2006). Tucker’s congruence coefficient as a meaningful index of factor similarity. Methodology: Eur J Res Methods Behav Social Sci.

[109] Lorenzo-Seva U, Ferrando P (2006). Evaluating structural equation models with unobservable variables and measurement error. Behav Res Methods Instrum Comput.

[110] Comrey AL, Lee HB. A first course in factor analysis. Psychology press; 2013.

[111] Worthington RL, Whittaker TA (2006). Scale development research: a content analysis and recommendations for best practices. Couns Psychol.

[112] Clark L, Watson D (1995). Construct validity: basic issues in objective scale development. Psychol Meas.

[113] Hair JF. Multivariate data analysis. 2009.

[114] Timmerman ME, Lorenzo-Seva U (2011). Dimensionality assessment of ordered polytomous items with parallel analysis. Psychol Methods.

[115] Yu C, Muthen B. Evaluation of model fit indices for latent variable models with categorical and continuous outcomes. In: *Paper presented at the annual conference of the American Educational Research Association, April 4, 2002, New Orleans: 2002*; 2002.

[116] Tie B, Chen G, He J (2022). Validation of the inflexible eating questionnaire in a large sample of chinese adolescents: psychometric properties and gender-related differential item functioning. Eat Weight Disorders-Studies Anorexia Bulimia Obes.

[117] Mundfrom DJ, Shaw DG, Ke TL (2005). Minimum sample size recommendations for conducting factor analyses. Int J Test.

[118] Hu Lt, Bentler PM (1999). Cutoff criteria for fit indexes in covariance structure analysis: conventional criteria versus new alternatives. Struct equation modeling: multidisciplinary J.

[119] Chen FF (2007). Sensitivity of goodness of fit indexes to lack of measurement invariance. Struct equation modeling: multidisciplinary J.

[120] Vadenberg R, Lance C (2000). A review and synthesis of the measurement in variance literature: suggestions, practices, and recommendations for organizational research. Organ Res Methods.

[121] Cheung GW, Rensvold RB (2002). Evaluating goodness-of-fit indexes for testing measurement invariance. Struct Equ Model.

[122] Dunn TJ, Baguley T, Brunsden V (2014). From alpha to omega: a practical solution to the pervasive problem of internal consistency estimation. Br J Psychol.

[123] McNeish D (2018). Thanks coefficient alpha, we’ll take it from here. Psychol Methods.

[124] Hair JF Jr, Sarstedt M, Ringle CM, Gudergan SP. Advanced issues in partial least squares structural equation modeling. saGe publications; 2017.

[125] Cohen J. Quantitative methods in psychology: a power primer. In: *psychological bulletin: 1992*. Citeseer; 1992.

[126] Salih M, Landers T (2019). The concept analysis of stigma towards chronic illness patient. Hospice and Palliative Medicine International Journal.

[127] Millen N, Walker C (2001). Overcoming the stigma of chronic illness: strategies for normalisation of a ‘spoiled identity’. Health Sociol Rev.

[128] Use omega rather than Cronbach’s alpha for estimating reliability. But…. *Communication Methods and Measures* 2020, 14(1):1–24.

[129] Tsang S, Royse CF, Terkawi AS (2017). Guidelines for developing, translating, and validating a questionnaire in perioperative and pain medicine. Saudi J Anaesth.

[130] Vogel DL, Wade NG, Haake S (2006). Measuring the self-stigma associated with seeking psychological help. J Couns Psychol.

[131] Vogel DL, Wade NG, Hackler AH (2007). Perceived public stigma and the willingness to seek counseling: the mediating roles of self-stigma and attitudes toward counseling. J Couns Psychol.

[132] Möller-Leimkühler AM (2002). Barriers to help-seeking by men: a review of sociocultural and clinical literature with particular reference to depression. J Affect Disord.

[133] Ongtengco N, Thiam H, Collins Z, De Jesus EL, Peterson CE, Wang T, Hendrix E, Ndiaye Y, Gueye B, Gassama O (2020). Role of gender in perspectives of discrimination, stigma, and attitudes relative to cervical cancer in rural Sénégal. PLoS ONE.

[134] Mugoya GC, Ernst K (2014). Gender differences in HIV-related stigma in Kenya. AIDS Care.

[135] Daley EM, Vamos CA, Wheldon CW, Kolar SK, Baker EA (2015). Negative emotions and stigma associated with a human papillomavirus test result: a comparison between human papillomavirus–positive men and women. J Health Psychol.

[136] Bagozzi RP (1981). Evaluating structural equation models with unobservable variables and measurement error: a comment.

